# Soil Organic Carbon Mineralization and Its Temperature Sensitivity under Different Substrate Levels in the Mollisols of Northeast China

**DOI:** 10.3390/life12050712

**Published:** 2022-05-10

**Authors:** He Yu, Yueyu Sui, Yimin Chen, Tianli Bao, Xiaoguang Jiao

**Affiliations:** 1 Heilongjiang Provincial Key Laboratory of Ecological Restoration and Resource Utilization for Cold Region, College of Modern Agriculture and Ecological Environment, Heilongjiang University, Harbin 150080, China; yuhehlju@163.com; 2 Key Laboratory of Mollisols Agroecology, Northeast Institute of Geography and Agroecology, Chinese Academy of Sciences, Harbin 150081, China; suiyy@iga.ac.cn (Y.S.); chenyimin@iga.ac.cn (Y.C.); 3 University of Chinese Academy of Sciences, Beijing 100049, China

**Keywords:** substrate quality, carbon dioxide, Kinetic theory, Mollisols, temperature sensitivity

## Abstract

Soil organic carbon (SOC) mineralization plays an important role in global climate change. Temperature affects SOC mineralization, and its effect can be limited by the substrate available. However, knowledge of the effects of temperature and substrate quality on SOC mineralization in the Mollisols of Northeast China is still lacking. In this study, based on a spatial transplant experiment, we conducted a 73-day incubation to examine the effects of temperature on SOC mineralization and its temperature sensitivity under different carbon levels. We found that the SOC content, incubation temperature and their interaction had significant effects on SOC mineralization. A higher SOC content and higher incubation temperature resulted in higher SOC mineralization. The temperature sensitivity of SOC mineralization was affected by the substrate quality. The temperature sensitivity of SOC mineralization, showed a downward trend during the incubation period, and the range of variation in the Q_10_ declined with the increment in the SOC content. The study suggested that there was a higher SOC mineralization in high levels of substrate carbon when the temperature increased. Further, SOC mineralization under higher SOC contents was more sensitive to temperature changes. Our study provides vital information for SOC turnover and the CO_2_ sequestration capacity under global warming in the Mollisols of Northeast China and other black soil regions of the world.

## 1. Introduction

Soil organic carbon (SOC) is one of the most important carbon pools in terrestrial ecosystems, accounting for 60~80% of the global terrestrial carbon [1]. SOC decomposition in soils affects atmospheric CO_2_ concentrations [2] and subsequently influences global climate change [3]. The study of SOC decomposition has been the focus of attention worldwide. Additionally, terrestrial ecosystems and their carbon dynamics significantly impact the global carbon budget [4]. Most studies have suggested that the changes in soil carbon content are associated with different types of land use [5,6] and different soil moisture and temperature conditions [7,8]. Better understanding of the dynamics of terrestrial SOC in different ecosystems is essential to determine SOC decomposition and turnover. The quantitative dynamics of SOC are important for predicting ecological processes and assessing soil fertility.

SOC mineralization, which mediates critical ecosystem processes important for the **decomposition** **and turnover** of organic matter, is an important monitor of the soil carbon cycle and can provide an estimate of soil carbon decomposition [9]. Carbon mineralization can be affected by many factors, such as **soil organic matter (SOM) quality and quantity, oxygen (O_2_)**, water availability, soil biota and soil temperature [10]. One of the most important drivers affecting SOC mineralization is soil temperature, which might be considered a driver of organic matter turnover in soils [1]. The temperature dependence of SOC mineralization, which is the key source of soil heterotrophic respiration, has been the subject of intense scientific debate [8]. Liu et al. [11] suggested that elevated temperature resulted in an exponential reduction in dissolved organic carbon (DOC). Xiao et al. [12] found that rising temperatures tended to result in a higher portion of stable C in soils. The observed contradictory views indicate that the effects of soil temperature on SOC are complex. The determination of SOC mineralization under different soil temperatures may provide more insights into the causal processes of global warming.

Kinetic theory indicates that the temperature sensitivity of SOM decomposition should increase with substrate recalcitrance [13]. Carney et al. [14] have suggested that carbon inputs stimulate microbial activity and result in higher SOC turnover. Giardina and Ryan [15] found that SOC mineralization in mineral soils was controlled more by substrate quality than temperature. A study on Mollisols demonstrated that when incubated at 25 °C, soils with a higher SOC content had higher CO_2_ production, but when incubated at 15 °C, no link between the SOC content and CO_2_ production was detected [16]. Substrate quality and availability affect the decomposition of SOM [17], and soil temperature influences SOC mineralization by its effects on microbial metabolic activity. The combination of the above two drivers has a interactive effect on SOC turnover rates compared with either factor alone [18]. Thus, the warming effect may be limited by the amount of substrate available and water for decomposition [19]. A better understanding of SOC mineralization is needed to assess the effects of soil temperature and substrate inputs on soil carbon storage.

Northeast China is one of the major regions of Mollisols worldwide. Mollisols, which have a large C pool, are characterized by high SOC and nutrient contents [20]. High amounts of CO_2_ can be released from the carbon in black soil. Differences in the SOC density and structure among different soil types result in the characteristics of SOC mineralization and its temperature sensitivity [21]. Therefore, increased recognition of regional SOC mineralization and its temperature sensitivity are important for understanding the C cycle as well as for improving C management strategies in the Mollisols of Northeast China [6,7].

Based on a spatial transplant experiment, we conducted an incubation experiment to examine how substrate C contents affect SOC mineralization under different temperatures and investigate its temperature sensitivity (Q_10_). We hypothesized that higher SOC content would induce larger CO_2_ production than those low levels of SOC content with the increment of temperature. In order to test the hypothesis, our objectives were to (1) examine how temperature affects SOC mineralization; (2) evaluate the effect of different SOC contents on SOC stability; and (3) determine the temperature sensitivity of SOC mineralization under substrates with different C contents. This study is necessary for determining SOC stocks and the CO_2_ sequestration potential in response to global warming in Mollisols of Northeast China and other black soil regions of the world.

## 2. Materials and Methods

### 2.1. Study Region

In October 2004, five sampling sites in Northeast China were selected for this study based on the SOC content. These sites were located in Lishu County and Dehui city in Jilin Province and in Hailun city, Bei’an city, and Nenjiang County in Heilongjiang Province in China. These regions have a typical temperate continental monsoon climate. The soil is classified as typical black soil, i.e., a Mollisol according to USDA soil taxonomy. Soil inorganic C could be ignored due to the lack of carbonates in these soils. Maize (*Zea mays*) is a common cultivated crop in these regions.

### 2.2. Experimental Design

We collected soil samples from a size of 1.4 m (length) × 1.2 m (width) × 1.0 m (depth) by wooden boxes from Lishu County, Dehui city, Hailun city, Bei’an city and Nenjiang County. These samples were transported to the Hailun Agroecology Experiment Station of the Chinese Academy of Sciences. The mean annual temperature is 1.5 °C in this region, and the mean annual precipitation ranges from 500 mm to 600 mm. With the use of a spatial transplant method (The crops were planted in a unified mode and management that eliminated the complexity of different climate conditions and land management ways), same standard (1.4 m × 1.2 m × 1.0 m) plots were established. The plots were separated by a 20-cm-thick brick wall, which was covered with cement and pasted with tarpaulin inside to minimize the risk of sampling nonindependent areas. In the study, treatments were divided into SOC10, SOC19, SOC29, SOC34, and SOC63 according to the C contents in the soils from the above sampling regions. Each treatment consisted of three replicates. Site descriptions are shown in Table 1.

### 2.3. Soil Sampling and Preparation

The selected samples from different regions were used to determine the effect of carbon levels on SOC mineralization. Clearly, under the premise of satisfying different C contents, the other physical-chemical properties of the soil samples were not consistent. In fact, the situation in the field was complex and changed. The samples, obtained from different regions, can better reflect the actual soil conditions in the field. A single-variable experiment is necessary to explore the complicated effects of the soil C level on SOC mineralization in the future.

After maize harvest in October 2016, twenty 10 cm × 5 cm × 20 cm (length × width × depth) samples were randomly collected to a depth of 0–20 cm at each sampling site. The twenty subsamples were thoroughly mixed to generate one composite sample, and fine roots and other residues were removed from the samples. The composite sample was the total amount of the twenty samples. Soon after collection, the samples were transported to the Key Laboratory of Ecological Restoration and Resource Utilization for Cold Region in Harbin, Heilongjiang Province. All samples were air-dried and sieved through a 1 mm mesh for the incubation experiment, the measurement of soil physical and chemical properties. The rest of the samples were sieved through a 0.25 mm mesh to determine SOC, total nitrogen (TN), total phosphorus (TP) and total potassium (TK). Soil pH was determined with an automatic acid-base titrator using a 1:2.5 soil:water suspension. The SOC content was measured by the Walkley Black method [22]. TN was determined by the kjeldahl method. TP was determined via the H_2_SO_4_-HClO_4_ digestion method. TK was analyzed according to NaOH melting with flame photometry. Available nitrogen (AN) was measured by alkali hydrolysis diffusion method. Available phosphorus (AP) was determined using the NaHCO_3_ leaching molybdenum antimony colorimetric technique, and available potassium (AK) was tested by NH_4_OAc extraction with flame photometry [23]. Soil properties are presented in Table 2.

### 2.4. Laboratory Incubation Experiment

We incubated soils in the laboratory using a factorial design of substrate (5 levels: SOC10, SOC19, SOC29, SOC34, and SOC63) and temperature (4 levels: 5 °C, 15 °C, 25 °C, and 35 °C) with three replicates. Air-dried soils (100 g) were placed in a 1000 mL jar and preincubated at 60% water-holding capacity (WHC) at 25 °C in the dark for 7 days. The WHC was determined by the method described by Alef and Nannipieri (1995) [24]. After preincubation, the samples were incubated at selected temperatures (5, 15, 25 or 35 °C) in the dark for 73 days. It is worth noting that the changes in the indices in the study tended to be stable after 73 days of incubation. During the incubation, samples were sealed in the incubator, and soil moisture was adjusted to 60% of WHC with deionized water.

### 2.5. CO_2_ Production

In this study, the accumulation of CO_2_ production was used to represent SOC mineralization in the soils. A beaker containing 25 mL 1.0 mol L^−1^ NaOH solution was placed in each jar to capture evolved CO_2_. The NaOH solution was exchanged on days 1, 3, 5, 7, 14, 21, 28, 35, 42, 49, 56, 63, and 73 of the incubation. Then, the CO_2_ was titrated with 0.5 mol L^−1^ HCl in excessive amounts of BaCl_2_ [25]. We calculated the cumulative CO_2_ production, which characterized SOC mineralization, during two successive sampling intervals. The cumulative CO_2_ production was expressed as mg CO_2_ kg^−1^ soil, representing the amount of CO_2_ released from the soil.

### 2.6. Evaluations and Calculations

In our study, the SOC mineralization rate and the temperature sensitivity of SOC mineralization were expressed as *Rs* and *Q*_10_ [26], respectively. The following equations were used.

(1) $$R_{s}=a\cdot e^{bT}$$

(2) $$Q_{10}=e^{10b}$$

where $R_{s}$ is the SOC mineralization rate (mg SOC kg^−1^ soil d^−1^), T represents the incubation temperature, a is the SOC mineralization rate at a temperature of 0 °C, and b is the temperature coefficient, which is related to the *Q*_10_ (increase in the rate of respiration over a 10 °C increase in temperature).

### 2.7. Data Analysis

The Kolmogorov-Smirnov test and Levene’s test were used to determine the normality and equality of the data, respectively. One-way analysis of variance (ANOVA) and least significant difference (LSD) multiple comparisons (*p* < 0.05) were used to assess the soil physical-chemical properties, soil cumulative CO_2_ production and *Q*_10_. Two-way ANOVA was performed to evaluate the effects of SOC level and soil temperature on cumulative CO_2_ production. The relationship between cumulative CO_2_ production and selected soils was evaluated with Pearson correlation analysis. The effects of the interaction of SOC contents and incubation temperatures on cumulative CO_2_ production were determined by a univariate general linear model with Duncan’s test. Curve estimation with an exponential model was used to evaluate parameter *b* in Equation (1). All statistical analyses were performed using SPSS statistical software ver. 20 (SPSS Inc., Chicago, IL, USA). Graphs were generated using SigmaPlot 12.5.

## 3. Results

### 3.1. Changes in Cumulative CO_2_ Production under Different SOC Contents at 5, 15, 25 and 35 °C

**Figure 1:**
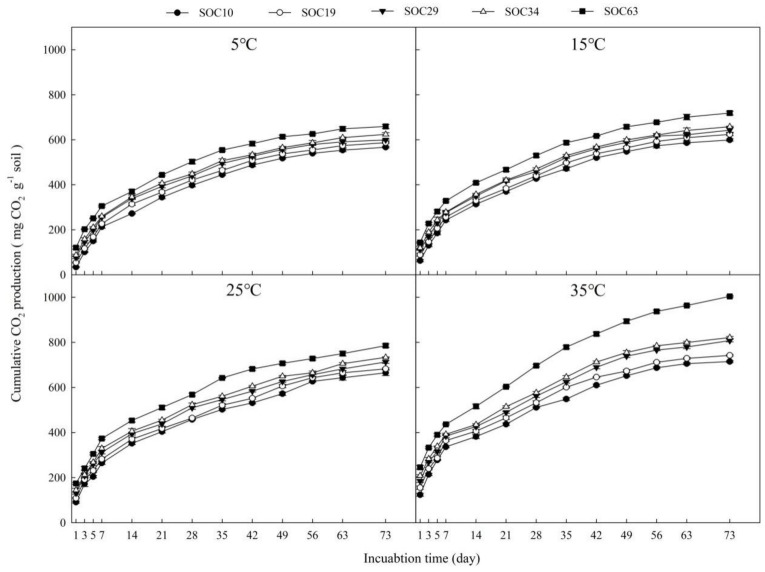
Changes in cumulative CO_2_ production throughout the incubation at different soil temperatures (5 °C, 15 °C, 25 °C and 35 °C). Data scatters are means ± standard error (*n* = 9).

The cumulative CO_2_ production from soils with different SOC contents under different incubation temperatures can be seen as Figure 1, and the cumulative CO_2_ production ranged from 567.10 to 1003.91 mg CO_2_ kg^−1^ soil at the end of the incubation. During the incubation period, we found the cumulative CO_2_ production increased with the increase in the SOC content. After 7 days of incubation, the cumulative CO_2_ production under different SOC treatments began to show obvious differences as follows: SOC63 > SOC34 > SOC29 > SOC19 > SOC10. The cumulative CO_2_ production stabilized across the treatments at different temperatures after 49 days of incubation. We also found that cumulative CO_2_ production increased as the incubation temperature increased, with significantly higher cumulative CO_2_ production at higher temperatures with increasing SOC content (Supplementary Tables S1–S9).

### 3.2. Characteristics of the Q_10_ Value under Different SOC Contents

**Figure 2:**
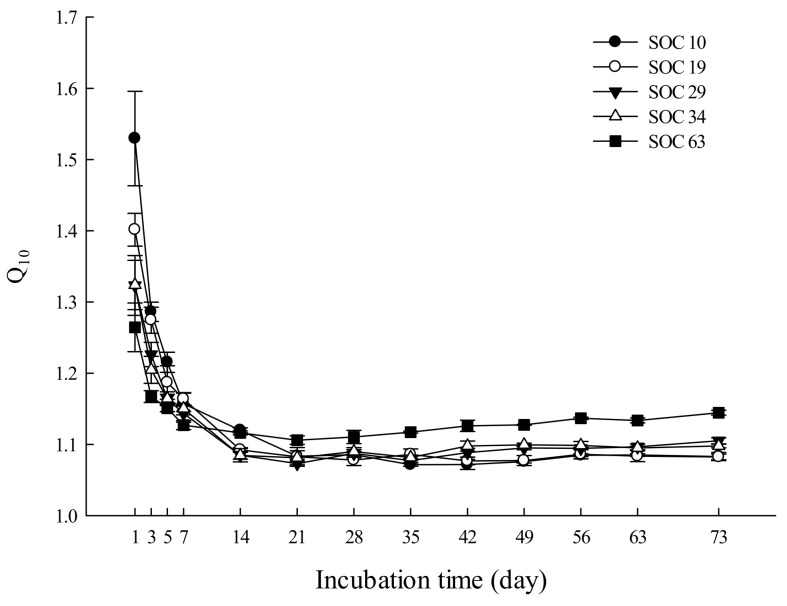
Effects of the soil carbon content on the *Q*_10_ value. Data scatters are means ± standard error (*n* = 9).

We averaged the *Q*_10_ values from the different incubation temperatures to reflect the temperature sensitivity of SOC mineralization in the study, and we found that the *Q*_10_ value showed a descending trend with the extension of the incubation time (Figure 2). During the incubation, the *Q*_10_ value varied in response to different SOC contents, ranging from 1.07 to 1.53, 1.08~1.40, 1.07~1.32, 1.08~1.32, and 1.11~1.26 in SOC10, SOC19, SOC29, SOC34 and SOC63, respectively.

**Figure 3:**
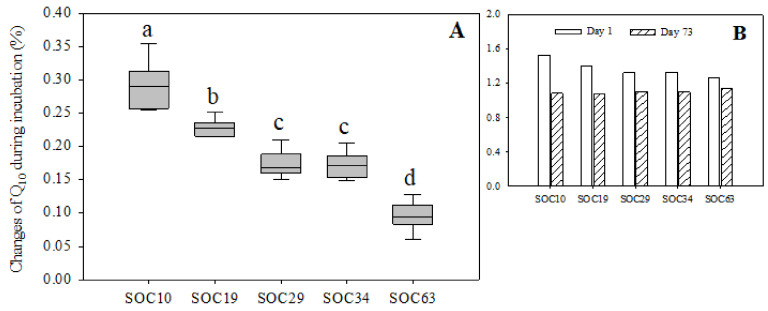
Changes in the *Q*_10_ value under different carbon levels during the incubation. Vertical bars of (**A**) = (Value of *Q*_10_ on the first day of incubation−value of *Q*_10_ on the 73rd day of incubation)/value of *Q*_10_ on the first day of incubation. (**B**) Day 1 = Value of *Q*_10_ on the first day of incubation; Day 73 = Value of *Q*_10_ on the 73rd day of incubation. Different lowercase letters indicate significant differences between SOC levels.

Both the highest and lowest *Q*_10_ values appeared in SOC10 on day 1 and day 35, respectively. The *Q*_10_ value showed a downward trend with little fluctuation and roughly leveled off by the end of the incubation in SOC10, SOC19 and SOC34. In SOC29 and SOC63, the *Q*_10_ value continued to decrease until day 21 and showed a modest upward trend with fluctuation thereafter. During the first five days of the incubation, soils with lower SOC contents had higher *Q*_10_ values. At the end of the incubation, the *Q*_10_ value in descending order was SOC63, SOC29, SOC34, SOC10 and SOC19 (Supplementary Table S10). Additionally, we also examined the changes in the *Q*_10_ value between the first day and the end of the incubation (the 73rd day of the incubation), and we found that the range of variation in the *Q*_10_ value decreased with the increase in the SOC content. There were significant differences of the *Q*_10_ between SOC63 and other treatments (Figure 3 and Supplementary Table S10).

### 3.3. Key Factors That Drive Changes in SOC Mineralization

According to the two-way ANOVA, we found that the SOC contents, incubation temperature and their interactions significantly affected SOC mineralization, and the temperature had a larger effects on SOC mineralization than the SOC contents (Table 3). Additionally, Pearson correlation analysis showed that soil organic carbon, total N, total P, total K, available N and available K were significantly positively correlated with cumulative CO_2_ production, indicating that soil nutrients were the main factor that determined the changes in SOC mineralization (Table 4).

## 4. Discussion

### 4.1. Effects of SOC Contents and Temperature on SOC Mineralization

The temperature effect may be limited by the substrate available for decomposition [19]. In this study, we found that the temperature and substrate C contents had significant effects on cumulative CO_2_ production (Figure 1; Table 3).

A temperature increase resulted in a higher cumulative CO_2_ production (Figure 1). Many incubation experiments have shown that increasing temperature can promote SOC mineralization [17,27,28]. Temperature affects SOC mineralization by its effects on microbial metabolic activity. An increase in temperature is favorable to microbial activity and therefore increased the turnover rate of SOC [29,30]. Leifeld and Fuhrer [28] also suggested that temperature is a major controlling factor for SOC turnover. The responses of SOC to temperature are important for the evaluation of possible atmospheric feedbacks from the SOM reservoir [31]. In the current study, the incubation environment was not suitable for the collection of microbial data. Additional research needs to be conducted to explore how changes in the microbial community drive variations in SOC mineralization.

As expected, we found that the cumulative CO_2_ production increased with increasing substrate carbon contents (Figure 1), and the alterations in cumulative CO_2_ production were significantly correlated with soil nutrients (Table 4). Substrate quality affected cumulative CO_2_ production, with the magnitudes varying across soils and substrate SOC [16]. There are higher C mineralization rates in the soils have been found in high contents of SOC [32]. Additionally, these results may primarily be attributed to microbial effects. Many studies have indicated that changes in soil nutrients modify microbial growth [33,34,35]. Greater C content lead to an increase in the abundance of microorganisms. A larger microbial community may be more efficient at SOC decomposition [14,15]. Competition is less crucial to limiting soil microbes because more niches may appear on account of improvements in soil nutritional resources [36]. Thus, the high levels of carbon may stimulate microbial activity and result in higher SOC turnover. As a result, the increase in substrate carbon might enhance SOC mineralization.

We found the increased SOC contents and warming had strong effects on SOC mineralization, and these effects were pronounced when warming and substrate treatments were applied together (Figure 1, Table 3). These two drivers has a different effect on SOC mineralization has a different effect on than either treatment alone [37]. Increased rates of SOC cycling caused by increased C inputs were exacerbated by warming [17]. It may be that warming affected the SOC turnover rate, the amount of CO_2_ production is determined by the amount of substrate C in soils [38]. Steinweg et al. [39] demonstrated that there are distinct mechanisms by which the temperature and substrate quality affect microbial respiration. The increased temperature promoted microorganisms to take up and metabolize substrates more quickly, and higher substrate C made greater amounts of C available to soil microorganisms in general. Furthermore, many other factors can influence the SOC mineralization, such as oxygen (O_2_), soil moisture and the fraction of substrate C and so on. Further research is necessary to explore the mechanism of these factors on SOC mineralization. 

### 4.2. Influences of SOC Contents on the Temperature Sensitivity of SOC Mineralization

Temperature sensitivity of SOC mineralization (*Q*_10_) in all carbon treatments showed a downward trend as incubation times increased, and a higher carbon content resulted in a smaller drop in *Q*_10_ in this study (Figure 2). The reason for the decline in *Q*_10_ may be the reduction in the SOC mineralization rate over time. Reichstein et al. [40] suggested that *Q*_10_ changed with incubation time, which may be caused by the alteration of labile C and recalcitrant C during incubation. Generally, the stability of SOC play a key role on the temperature sensitivity of the SOC decomposition, additional research needs to be conducted to explore whether changes in labile C and recalcitrant C drive changes in the SOC decomposition. 

To further determine the changes in *Q*_10_, we examined the variation amplitude of *Q*_10_ between the first day and the end of the incubation (the 73rd day of the incubation), and we found that *Q*_10_ was higher under a high carbon content than under low levels of carbon at the end of the incubation (Figure 3). The availability of SOC affects the response of soil heterotrophic respiration to temperature change, and *Q*_10_ decreases with the reduction in labile SOC content in soils [41]. The availability of soil nutrients was relatively high under the high soil carbon content compared with the low levels of soil carbon when the external temperature increased.

We also demonstrated that SOC mineralization under higher SOC contents was more sensitive to temperature changes in the middle and later period of SOC mineralization, and SOC mineralization under lower SOC contents was more sensitive to temperature changes at the early stage of SOC mineralization. To be degree, high levels of substrate carbon can stimulate SOC mineralization and result in greater SOC turnover. These findings can provide theoretical support for the impacts of different substrate gradients on SOC mineralization. Determining the temperature sensitivity of the decomposition of the different levels of SOC pools is critical for predicting the long-term impacts of climate change on SOC storage of Mollisols in Northeast China in the context of global warming. 

## 5. Conclusions

In the study, the cumulative CO_2_ production was relatively high under the high levels of soil carbon content. It suggested that high contents of SOC in soils resulted in a higher C mineralization. Changes in temperature can also affect the SOC mineralization. Larger cumulative CO_2_ production were found with the increasing of temperature. We also found the effects of increased SOC contents and warming on SOC mineralization were significant when these two drivers were applied together. High levels of substrate carbon can stimulate SOC mineralization and result in greater SOC turnover when the temperature increased. Q_10_, which represents the temperature sensitivity of SOC mineralization, varied between different SOC contents. The results suggested that the SOC mineralization under higher SOC contents was more sensitive to temperature changes. These fndings are important for achieving a better understanding of SOC turnover and the CO_2_ sequestration capacity under global warming in the Mollisols of Northeast China and other black soil regions of the world.

## Figures and Tables

**Table 1:** Sampling site descriptions.

Site	Coordinates	Mean Annual Temperature (°C)	Mean Annual Precipitation (mm)	Crop
Lishu	N 43°20′, E 124°28′	5.4	556.2	Maize
Dehui	N 44°12′, E 125°33′	4.5	457.6	Maize
Hailun	N 47°27′, E 126°56′	1.5	549.3	Maize
Bei’an	N 48°09′, E 126°44′	1.1	523.4	Maize
Nenjiang	N 49°08′, E 125°37′	−0.2	532.1	Maize

**Table 2:** Soil properties of selected sites.

Site	SOC (g kg^−1^)	TN (g kg^−1^)	TP (g kg^−1^)	TK (g kg^−1^)	AN (mg kg^−1^)	AP (mg kg^−1^)	AK (mg kg^−1^)	pH	C/N
Lishu	9.63 ± 2.44 a	0.79 ± 0.03 a	0.64 ± 0.02 a	12.58 ± 1.69	97.39 ± 9.88 a	64.10 ± 8.98 a	145.11 ± 10.99	6.63 ± 0.12 a	12.21 ± 0.89
Dehui	18.56 ± 2.67 b	1.68 ± 0.04 b	0.84 ± 0.01 a	12.97 ± 1.27	120.73 ± 14.87 a	26.29 ± 7.31 b	151.24 ± 9.54	5.95 ± 0.06 b	11.05 ± 1.19
Hailun	29.35 ± 3.56 c	2.55 ± 0.11 c	1.60 ± 0.03 b	13.84 ± 2.76	218.00 ± 25.98 b	57.81 ± 4.77 c	156.77 ± 15.33	6.10 ± 0.02 b	11.48 ± 2.18
Bei’an	34.11 ± 2.98 c	2.86 ± 0.08 c	1.93 ± 0.07 bc	14.37 ± 2.38	338.74 ± 19.87 c	58.34 ± 3.99 c	167.88 ± 14.86	5.42 ± 0.11 c	11.87 ± 0.99
Nenjiang	63.17 ± 4.76 d	4.87 ± 0.13 d	2.43 ± 0.05 c	15.56 ± 3.04	366.72 ± 26.56 c	57.15 ± 4.68 c	163.44 ± 16.21	6.34 ± 0.05 a	12.99 ± 1.21

Note: Different lowercase letters indicate significant differences among sampled sites. Unmarked means that there are no significant differences between treatments. C/N = SOC: TN. Values are means ± standard error (*n* = 9).

**Table 3:** Effects of SOC level and soil temperature on cumulative CO_2_ production based on analysis of variance.

Index	Factors	F Value	df	*p*
CO_2_ production	C level	380.25	22	*p* < 0.01
	Soil temperature	1126.20	28	*p* < 0.01
	C level × Soil temperature	35.30	19	*p* < 0.01

Note: Data for statistical analysis are based on a *n* = 12 samples per treatment.

**Table 4:** Correlations between soil physical-chemical properties and cumulative CO_2_ production.

	SOC	TN	TP	TK	AN	AP	AK	pH	C/N
Pearson Correlation	0.936 **	0.927 **	0.847 **	0.915 **	0.896 **	0.194	0.761 *	−0.106	0.331
Sig. (2-tailed)	<0.001	<0.001	<0.001	<0.001	<0.001	0.241	0.021	0.389	0.281

Note: Data for statistical analysis are based on a *n* = 12 samples per treatment. * and ** represent significance differences at *p* < 0.05 and *p* < 0.01, respectively.

## Data Availability

The datasets generated and analyzed during the current study are available from the corresponding author on reasonable request.

## Supplementary Materials

The following supporting information can be downloaded at: https://www.mdpi.com/article/10.3390/life12050712/s1. Table S1. Changes in cumulative CO_2_ production with varied SOC contents when incubated at 5 °C. Table S2. Variations of cumulative CO_2_ production among different SOC contents when incubated at 15 °C. Table S3. Alterations in cumulative CO_2_ production between SOC contents when incubated at 25 °C. Table S4. Changes in cumulative CO_2_ production with different SOC contents when incubated at 35 °C. Table S5. Alterations of cumulative CO_2_ production under SOC10 among incubation temperatures. Table S6. Changes in cumulative CO_2_ production in the SOC19 with different incubation temperatures. Table S7. Variations of cumulative CO_2_ production under SOC29 between incubation temperatures. Table S8. Changes in cumulative CO_2_ production under SOC34 with incubation temperatures. Table S9. Variations of cumulative CO_2_ production in the SOC63 among incubation temperatures. Table S10. Changes in the *Q*_10_ values with SOC contents in soils.
