# Supplementary material for: Soil Organic Carbon Mineralization and Its Temperature Sensitivity under Different Substrate Levels in the Mollisols of Northeast China
Source: Life (Basel). 2022 May 10;12(5):712. doi: 10.3390/life12050712 (PMC9143832; doi:10.3390/life12050712)
Supplement: Supplementary file 1 [file life-12-00712-s001.zip › life-1689241-supplementary.pdf]

Soil organic carbon mineralization and its temperature sensitivity under different carbon contents in the Mollisols of Northeast China

Supplementary Table S1. Changes in cumulative CO_2 production with varied SOC contents when incubated at 5 °C.

Incubation time	SOC10		SOC19		SOC29		SOC34		SOC63	
1	34.32	Dl	54.09	Ck	76.94	Bl	87.52	Bl	120.48	Ak
3	101.30	Ek	116.88	Dj	146.16	Ck	158.73	Bk	202.55	Aj
5	150.23	Dj	168.26	Ci	198.47	Bj	211.08	Bj	250.97	Ai
7	213.18	Di	226.88	Ch	255.76	Bi	260.04	Bi	305.55	Ah
14	272.10	Dh	314.25	Cg	338.50	Bh	346.37	Bh	370.33	Ag
21	344.36	Dg	367.53	Cf	393.25	Bg	407.01	Bg	444.25	Af
28	397.78	Df	421.20	Ce	439.72	BCf	448.62	Bf	502.92	Ae
35	444.63	Ce	463.87	Cd	494.93	Be	507.34	Be	554.44	Ad
42	487.66	Cd	508.70	BCc	526.87	Bd	533.66	Bd	582.80	Ac
49	518.10	Dc	537.41	Cb	557.29	Bc	565.34	Bc	613.74	Ab
56	539.76	Cb	554.97	Cb	578.73	Bb	586.72	Bb	626.07	Ab
63	553.90	Da	574.25	CDa	590.85	BCab	609.54	Ba	648.71	Aa
73	567.10	Da	587.11	Ca	599.33	Ca	624.05	Ba	659.32	Aa

Note: Values correspond to averages of n = 3 samples per treatment. Different uppercase letters indicate significant differences between SOC contents at the same incubated time, and different lower letters indicate significant differences between incubated time at the same SOC contents ($p < 0.05$).

Supplementary Table S2. Variations of cumulative CO_2 production among different SOC contents when incubated at 15 °C.

Incubation time	SOC10		SOC19		SOC29		SOC34		SOC63	
1	63.01	Dl	89.03	Cl	111.97	Bl	119.41	Bl	143.33	Ak
3	130.48	Dk	146.60	Dk	170.07	Ck	189.63	Bk	228.08	Aj
5	185.42	Dj	206.61	CDj	230.81	BCj	243.47	Bj	281.01	Ai
7	242.81	Ci	256.71	Ci	276.93	Bi	278.68	Bi	328.60	Ah
14	313.79	Dh	329.73	Ch	349.63	Bh	357.55	Bh	409.09	Ag
21	369.90	Cg	383.47	Cg	416.81	Bg	420.57	Bg	466.99	Af
28	427.39	Cf	440.23	Cf	459.32	Bf	470.59	Bf	529.99	Ae
35	471.76	De	497.66	Ce	521.18	Be	529.71	Be	587.37	Ad
42	520.18	Dd	538.65	Cd	560.27	Bd	567.59	Bd	617.21	Ac
49	547.85	Cc	564.44	Cc	588.55	Bc	599.29	Bc	657.80	Ab
56	573.21	Db	592.97	Cb	616.04	Bb	621.01	Bb	677.57	Ab
63	586.65	Dab	609.41	CDab	622.30	BCb	641.60	Bab	701.00	Aa
73	599.27	Da	624.27	Ca	642.79	BCa	657.97	Ba	718.82	Aa

Note: Values correspond to averages of n = 3 samples per treatment. Different uppercase letters indicate significant differences between SOC contents at the same incubated time, and different lower letters indicate significant differences between incubated time at the same SOC contents ($p < 0.05$). The same as below.

Supplementary Table S3. Alterations in cumulative CO_2 production between SOC contents when incubated at 25 °C.

Incubation time	SOC10		SOC19		SOC29		SOC34		SOC63	
1	91.20	Dl	108.81	Cl	131.05	BCm	144.64	Bl	174.23	Am
3	172.15	Ck	191.40	BCk	208.19	Bl	212.89	Bk	241.20	Al
5	204.03	Dj	231.21	Cj	259.40	BCk	268.09	Bj	305.50	Ak
7	264.66	Ci	282.14	Ci	312.01	Bj	329.54	Bi	373.48	Aj
14	351.79	Dh	370.62	CDh	394.66	BCi	406.06	Bh	453.52	Ai
21	403.76	Dg	417.62	CDg	437.87	BCh	454.84	Bg	510.97	Ah
28	457.70	Cf	464.00	Cf	509.74	Bg	523.70	Bf	567.86	Ag
35	502.46	De	521.12	Ce	546.15	Bf	560.96	Be	642.18	Af
42	531.52	Ed	551.79	Dd	583.61	Ce	605.60	Bd	681.88	Ae
49	572.03	Ec	606.12	Dc	626.40	Cd	649.66	Bc	707.24	Ad
56	627.15	Db	643.04	CDb	656.59	BCc	664.77	Bc	728.22	Ac
63	643.08	Db	665.00	CDa	682.41	BCb	705.48	Bb	750.19	Ab
73	664.74	Ca	682.59	Ca	713.03	Ba	733.55	Ba	785.44	Aa

Supplementary Table S4. Changes in cumulative CO_2 production with different SOC contents when incubated at 35 °C.

Incubation time	SOC10		SOC19		SOC29		SOC34		SOC63	
1	123.70	El	155.50	Dl	184.48	Cl	208.34	Bl	245.70	Am
3	213.93	Dk	239.65	Ck	269.45	BCk	283.74	Bk	332.87	Al
5	278.35	DEj	286.40	Dj	316.37	Cj	338.93	Bj	389.83	Ak
7	336.49	Di	364.37	Ci	384.15	BCi	392.13	Bi	436.29	Aj
14	382.10	Dh	405.83	CDh	426.36	BCh	435.14	Bh	516.22	Ai
21	436.72	Eg	465.39	Dg	489.38	Cg	514.52	Bg	603.17	Ah
28	511.17	Df	531.94	Cf	561.60	Bf	576.75	Bf	696.66	Ag
35	548.43	Ee	601.00	De	624.51	Ce	646.38	Be	778.82	Af
42	610.05	Ed	645.78	Dd	689.51	Cd	712.67	Bd	837.41	Ae
49	651.78	Cc	672.64	Cc	738.78	Bc	754.92	Bc	893.69	Ad
56	687.23	Eb	711.33	Db	765.75	Cb	784.64	Bb	937.01	Ac
63	705.46	Ea	728.87	Da	779.31	Cb	799.26	Bb	963.48	Ab
73	714.95	Ea	742.28	Da	807.80	Ca	821.50	Ba	1003.91	Aa

Note: Values correspond to averages of n = 3 samples per treatment. Different uppercase letters indicate significant differences between SOC contents at the same incubated time, and different lower letters indicate significant differences between incubated time at the same SOC contents ($p < 0.05$).

Supplementary Table S5. Alterations of cumulative CO_2 production under SOC10 among incubation temperatures.

Incubation temperature	5 °C		15 °C		25 °C		35 °C	
1	34.32	D	63.01	C	91.20	B	123.70	A
3	101.30	D	130.48	C	172.15	B	213.93	A
5	150.23	D	185.42	C	204.03	B	278.35	A
7	213.18	D	242.81	C	264.66	B	336.49	A
14	272.10	D	313.79	C	351.79	B	382.10	A
21	344.36	D	369.90	C	403.76	B	436.72	A
28	397.78	D	427.39	C	457.70	B	511.17	A
35	444.63	D	471.76	C	502.46	B	548.43	A
42	487.66	C	520.18	B	531.52	B	610.05	A
49	518.10	D	547.85	C	572.03	B	651.78	A
56	539.76	D	573.21	C	627.15	B	687.23	A
63	553.90	D	586.65	C	643.08	B	705.46	A
73	567.10	D	599.27	C	664.74	B	714.95	A

Note: Values correspond to averages of n = 3 samples per treatment. Different uppercase letters indicate significant differences between incubation temperatures at the same SOC contents ($p < 0.05$).

Supplementary Table S6. Changes in cumulative CO_2 production in the SOC19 with different incubation temperatures.

Incubation temperature	5 ℃		15 ℃		25 ℃		35 ℃	
1	54.09	D	89.03	C	108.81	B	155.50	A
3	116.88	D	146.60	C	191.40	B	239.65	A
5	168.26	C	206.61	B	231.21	B	286.40	A
7	226.88	D	256.71	C	282.14	B	364.37	A
14	314.25	C	329.73	C	370.62	B	405.83	A
21	367.53	C	383.47	C	417.62	B	465.39	A
28	421.20	C	440.23	C	464.00	B	531.94	A
35	463.87	D	497.66	C	521.12	B	601.00	A
42	508.70	C	538.65	B	551.79	B	645.78	A
49	537.41	D	564.44	C	606.12	B	672.64	A
56	554.97	D	592.97	C	643.04	B	711.33	A
63	574.25	D	609.41	C	665.00	B	728.87	A
73	587.11	D	624.27	C	682.59	B	742.28	A

Note: Values correspond to averages of n = 3 samples per treatment. Different uppercase letters indicate significant differences between incubation temperatures at the same SOC contents ($p < 0.05$).

Supplementary Table S7. Variations of cumulative CO_2 production under SOC29 between incubation temperatures.

Incubation temperature	5 ℃		15 ℃		25 ℃		35 ℃	
1	76.94	D	111.97	C	131.05	B	184.48	A
3	146.16	D	170.07	C	208.19	B	269.45	A
5	198.47	D	230.81	C	259.40	B	316.37	A
7	255.76	C	276.93	C	312.01	B	384.15	A
14	338.50	C	349.63	C	394.66	B	426.36	A
21	393.25	D	416.81	C	437.87	B	489.38	A
28	439.72	C	459.32	C	509.74	B	561.60	A
35	494.93	D	521.18	C	546.15	B	624.51	A
42	526.87	D	560.27	C	583.61	B	689.51	A
49	557.29	D	588.55	C	626.40	B	738.78	A
56	578.73	D	616.04	C	656.59	B	765.75	A
63	590.85	D	622.30	C	682.41	B	779.31	A
73	599.33	D	642.79	C	713.03	B	807.80	A

Note: Values correspond to averages of n = 3 samples per treatment. Different uppercase letters indicate significant differences between incubation temperatures at the same SOC contents ($p < 0.05$).

Supplementary Table S8. Changes in cumulative CO_2 production under SOC34 with incubation temperatures.

Incubation temperature	5 ℃		15 ℃		25 ℃		35 ℃	
1	87.52	D	119.41	C	144.64	B	208.34	A
3	158.73	D	189.63	C	212.89	B	283.74	A
5	211.08	D	243.47	C	268.09	B	338.93	A
7	260.04	D	278.68	C	329.54	B	392.13	A
14	346.37	C	357.55	C	406.06	B	435.14	A
21	407.01	C	420.57	C	454.84	B	514.52	A
28	448.62	D	470.59	C	523.70	B	576.75	A
35	507.34	D	529.71	C	560.96	B	646.38	A
42	533.66	D	567.59	C	605.60	B	712.67	A
49	565.34	D	599.29	C	649.66	B	754.92	A
56	586.72	D	621.01	C	664.77	B	784.64	A
63	609.54	D	641.60	C	705.48	B	799.26	A
73	624.05	D	657.97	C	733.55	B	821.50	A

Note: Values correspond to averages of n = 3 samples per treatment. Different uppercase letters indicate significant differences between incubation temperatures at the same SOC contents ($p < 0.05$).

Supplementary Table S9. Variations of cumulative CO_2 production in the SOC63 among incubation temperatures.

Incubation temperature	5 ℃		15 ℃		25 ℃		35 ℃	
1	120.48	D	143.33	C	174.23	B	245.70	A
3	202.55	C	228.08	B	241.20	B	332.87	A
5	250.97	D	281.01	C	305.50	B	389.83	A
7	305.55	D	328.60	C	373.48	B	436.29	A
14	370.33	D	409.09	C	453.52	B	516.22	A
21	444.25	D	466.99	C	510.97	B	603.17	A
28	502.92	D	529.99	C	567.86	B	696.66	A
35	554.44	D	587.37	C	642.18	B	778.82	A
42	582.80	D	617.21	C	681.88	B	837.41	A
49	613.74	D	657.80	C	707.24	B	893.69	A
56	626.07	D	677.57	C	728.22	B	937.01	A
63	648.71	D	701.00	C	750.19	B	963.48	A
73	659.32	D	718.82	C	785.44	B	1003.91	A

Note: Values correspond to averages of n = 3 samples per treatment. Different uppercase letters indicate significant differences between incubation temperatures at the same SOC contents ($p < 0.05$).

Supplementary Table S10. Changes in the Q_{10} values with SOC contents in soils.

Incubation time	SOC10		SOC19		SOC29		SOC34		SOC63	
1	1.5295	A	1.4015	AB	1.3232	B	1.3239	B	1.2644	B
3	1.2863	A	1.2745	AB	1.2264	BC	1.2048	CD	1.1672	D
5	1.2154	A	1.187	AB	1.1639	B	1.1643	B	1.1508	B
7	1.1572	AB	1.1638	A	1.1435	AB	1.1502	AB	1.1272	B
14	1.1198	A	1.0924	B	1.0848	B	1.0847	B	1.1163	A
21	1.0835	AB	1.0827	AB	1.0731	B	1.0813	AB	1.1061	A
28	1.0857	B	1.0782	B	1.0875	B	1.0900	AB	1.1105	A
35	1.0717	C	1.086	B	1.0773	BC	1.0816	BC	1.1173	A
42	1.0719	C	1.0769	BC	1.0885	BC	1.0978	B	1.1261	A
49	1.0759	C	1.0774	C	1.0951	B	1.0995	B	1.1275	A
56	1.0849	C	1.0862	BC	1.0946	BC	1.0987	B	1.1368	A
63	1.0852	B	1.0837	B	1.0967	B	1.0951	B	1.1337	A
73	1.0831	C	1.0825	C	1.1051	B	1.0979	B	1.1446	A

Note: Values correspond to averages of n = 3 samples per treatment. Different uppercase letters indicate significant differences between SOC contents ($p < 0.05$).
